# The Complete Mitochondrial Genome of the Rice Moth, *Corcyra cephalonica*

**DOI:** 10.1673/031.012.7201

**Published:** 2012-06-26

**Authors:** Yu-Peng Wu, Jie Li, Jin-Liang Zhao, Tian-Juan Su, A-Rong Luo, Ren-Jun Fan, Ming-Chang Chen, Chun-Sheng Wu, Chao-Dong Zhu

**Affiliations:** ^1^ Institute of Loess Plateau, Shanxi University, Taiyuan 030006, China; ^2^ Key Laboratory of Zoological Systematics and Evolution (CAS), Institute of Zoology, Chinese Academy of Sciences, Beijing 100101, China; ^3^ Institute of Fruit Trees, Shanxi Academy of Agricultural Sciences, 030031; ^4^ Plant Protection and Quarantine Station of Shanxi Province, Taiyuan 030001, China; ^5^ College of Life Sciences, Capital Normal University, Beijing, 100048, China; ^6^ Institute of Plant Protection, Shanxi Academy of Agricultural Sciences, 030031; ^7^ Shanxi Academy of Agricultural Sciences, 030031

**Keywords:** Galleriinae, mitogenome

## Abstract

The complete mitochondrial genome (mitogenome) of the rice moth, *Corcyra cephalonica* Stainton (Lepidoptera: Pyralidae) was determined as a circular molecular of 15,273 bp in size. The mitogenome composition (37 genes) and gene order are the same as the other lepidopterans. Nucleotide composition of the *C. cephalonica* mitogenome is highly A+T biased (80.43%) like other insects. Twelve protein-coding genes start with a typical ATN codon, with the exception of *coxl* gene, which uses CGA as the initial codon. Nine protein-coding genes have the common stop codon TAA, and the *nad2, cox1, cox2,* and *nad4* have single T as the incomplete stop codon. 22 tRNA genes demonstrated cloverleaf secondary structure. The mitogenome has several large intergenic spacer regions, the spacer1 between *trnQ* gene and *nad2* gene, which is common in Lepidoptera. The spacer 3 between *trnE* and *trnF* includes microsatellite-like repeat regions (AT)18 and (TTAT)_3_. The spacer 4 (16 bp) between *trnS2* gene and *nad1* gene has a motif ATACTAT; another species, *Sesamia inferens* encodes ATCATAT at the same position, while other lepidopteran insects encode a similar ATACTAA motif. The spacer 6 is A+T rich region, include motif ATAGA and a 20-bp poly(T) stretch and two microsatellite (AT)_9_, (AT)_8_ elements.

## Introduction

Animal mitogenomes are typically enclosed circular molecules of 14–20 kb in length with 37 genes, 13 protein-coding genes (PCGs), 22 transfer RNA (tRNA), and two ribosomal RNA (rRNA). It also contains an A+T rich non-coding area (also called control region) responsible for regulating transcription and replication of the mitogenome (Boore 1999; Taanman 1999). Mitogenomes have a simple structure, undergo fast evolution, are normally maternal inherited, and have been broadly applied in phylogenetic reconstruction, phylogeography, population structure and dynamics, and molecular evolution (Zhang et al. 1995; Nardi et al. 2003; Arunkumar et al. 2006). Recent advancements in sequencing technology have lead to rapid growth of mitogenome data in Genbank. To date, the complete mitogenome sequences of more than 140 species have been determined for insects, including 31 species of Lepidoptera that have been entirely or nearly entirely sequenced (Coates et al. 2005; Kim et al. 2006; Lee et al. 2006; Cameron et al. 2007; Cha et al. 2007; Cameron and Whiting 2008; Liu et al. 2008; Jiang et al. 2009; Hong et al. 2009; Pan et al. 2008; Salvato et al. 2008; Kim MI et al. 2009; Hu et al. 2010; Liao et al. 2010; Li et al. 2010; Zhao et al. 2010; Margam et al. 2011).

Lepidoptera is the second largest order after Coleoptera within in Insecta and includes moths and butterflies. Most of them are agricultural and forestry pests, pollinators, and resources insects (Li et al. 2009). *Corcyra cephalonica* Stainton (Lepidoptera: Pyralidae) is in a small subfamily of Galleriinae with 261 species of Pyralidae, which contains more than 330 species of 70 genera (Heppner 1991). The genus *Corcyra* contains only two species, *C. nidicolella* and *C. cephalonica;* the latter is known to be a stored product pest, and is controlled with botanical insecticides and trapped with sex pheromone (Türkera 1998; Allotey and Azalekor 2000; Coelho et al. 2007). *Corcyra cephalonica* is used as the host for cultivating *Trichogramma* and other parasitoid wasps (Muthukrishnan et al. 2003; Jalali et al. 2007). Moreover, it is lately being used as an experimental model insect. A group of the functional genes have been identified (Nagamanju et al. 2003; Chaitanya and Dutta-Gupta, 2010; Damara et al. 2010; Gullipalli et al. 2010), but information regarding the mitochondrial genome is lacking. The availability of the mitogenome sequence will definitely be beneficial in the basic and applied studies on *C. cephalonica.*

In this paper, the mitogenome of *C.* *cephalonica* was sequenced and analyzed. So far, there are four species within Pyraloidea with known mitogenome: *Diatraea saccharalis* (Li et al. 2010, *Ostriniafurnacalis* and *O. nubilalis* (Coates et al. 2005), and *Chilo suppressalis* [unpublished, JF339041].

## Materials and Methods

### DNA samples extraction

*Corcyra* eggs were collected from Guangdong Province of China and raised in the laboratory in Beijing. The hatched adults were collected, preserved in 100% ethanol, and stored at —20 °C. Total DNA was extracted and isolated from single specimens using the DNeasy Tissue kit (QIAGEN, www.qiagen.com) according to manufacturer instructions.

### Primer design, PCR, and sequencing

The short fragment amplifications were performed using the universal PCR primers from Simon et al. (1994). The degenerate and specific primer pairs were designed based on the known mitochondrial sequences in Lepidoptera, or designed by Primer 5.0 software on the fragments that we previously sequenced (Table 1). All the primers were synthesized by Shanghai Sangon Biotechnology Co., Ltd, www.sangon.com. For fragments of length less than 2 kb, PCR conditions were as follows: 95 °C for five min, 34 cycles of 94 °C for 30 sec, 50–55 °C (depending on primer combinations), 1–3 min (depending on putative length of the fragments) at 68 °C, and a final extension step of 72 °C for 10 min. For fragments of length longer than 2 kb, PCR conditions were as follows: 92 °C for two min, 40 cycles of 92 °C for 30 sec, 50–55 °C for 30 sec (depending on primer combinations), 60 °C for 12 min, and a final extension step of 60 °C for 20 min.

The entire mitogenome of the *Corcyra* was amplified in 17 fragments. For most fragments, 2× Taq PCR MasterMix (Tiangen Biotech Co., Ltd., www.tiangen.com) was used in the amplification; fragments longer than 2 kb (e.g., *rrnL-rrnS* and *nad4-cob)* and with higher AT contents (e.g., *rrnS-nad2* and *cox3-nad5*) were amplified using Takara LA Taq (Takara Co. www.takara-bio.com). All amplifications were performed on an Eppendorf Mastercycler and Mastercycler gradient in 50 µL reaction volumes. The reaction volume of 2 × Taq PCR MasterMix contained 22 µL sterilized distilled water, 25 µL 2× Master Mix, 1 µL of each primer (10 uM), and 1 µL of DNA template; the reaction volume of Takara LA Taq consisted of 26.5 µL sterilized distilled water, 5 µL 10×LA PCR Buffer II (Takara), 5 µL 25 mM MgCl_2_, 8 µL of dNTPs Mixture, 2 µL of each primer (10 µM), 1 µL of DNA template, and 0.5 µL (1.25 U) of Takara LA Taq polymerase (Takara).

The PCR products were detected via electrophoresis in 1% agarose gel, purified using the 3 S Spin PCR Product Purification Kit, and sequenced directly with ABI-377 automatic DNA sequencer. All fragments were sequenced from both strands. Short amplified products were sequenced directly by internal primers, and long amplified products were sequenced completely by primer walking. The *rrnS-nad2* region was sequenced after cloning. The purified PCR products were ligated to the *pEASY-T3* Cloning Vector (Beijing TransGen Biotech Co., Ltd., transgen.com.cn) and then sequenced by M13F and M13-R primers and walking. Sequencing was performed using ABI BigDye ver 3.1 dye terminator sequencing technology and run on ABI PRISM 3730×1 capillary sequencers.

### Analysis and annotation

Sequence annotation was performed using the DNAStar package (DNAStar Inc., www.dnastar.com). The sequence was checked manually for consistency by alignment, and tRNA genes were found using tRNAscan-SE software v. 1.21 (Lowe and Eddy 1997) with manual editing. The undermined putative tRNAs were identified by sequence alignment with other insects of Pyralidae (Diatraea, *O. furnacalis,* and *O. nubilalis)* using Bioedit (Hall 1999). Secondary structure was inferred using DNASIS v.2.5. The *trnS1(AGN)* secondarystructure was developed as proposed by Steinberg and Cedergren (1994). PCGs and rRNAs were identified by similarity to other lepidopteran sequences. The nucleotide sequences of the PCGs were translated based on the invertebrate mtDNA genetic code. Since the *Corcyra* does not utilize the AGG codon, use of the variant arthropod genetic code (Abascal et al. 2006) was unnecessary. Nucleotide composition and codon usage were calculated using MEGA4.0 (Tamura et al. 2007).

## Results

### Genome structure and organization

The *Corcyra* mitogenome is a circular molecule 15,273 bp in length; data were uploaded to Genbank (HQ897685). The *Corcyra* mitogenome showed the standard gene complement containing 13 PCGs, 2 rRNAs, 22 tRNAs, and non-coding regions typical for lepidopterans. The *trnM* is coded between the A+T rich region and tRNA-Ile (order is A+T *region-trnM-trnl-trnQ),* which was different from the ancestral gene order of insects (A+T *region-trnl-trnQ-trnM).* Since the *trnS2(UCN)* was not found by tRNAScan-SE, it was later determined by sequence comparison with other lepidopteran insects.

The *Corcyra* mitogenome was biased toward A+T content (80.43%) with the value falling into the lepidopteran range of 77.84% in *Ochrogaster lunifer* (Salvato et al. 2008) to 82.66% in *Coreana raphaelis* (Kim et al. 2006). Additionally, the A+T content was 78.96% in PCGs, 82.95%, in *rrnL* genes, and 85.86% in *rrns* genes. These values were also well within the range reported for other lepidopterans. The A+T content (96.58%) of A+T rich region was the highest value among the known lepidoteran MtDNA sequences (Table 3).

### Protein-coding genes

The initial and termination codons of thirteen PCGs are shown in Table 2. Twelve PCGs started with a typical ATN codon (ATT for *nad2, cox2, atp8, nad3, nad6;* ATA for *nad5, cob, nad1;* ATG for *atp6,cox3, nad4, nad4l).* One exception is cox*1*gene, which used CGA as a start codon.

The putative start codon CGA is common across insects (Fenn et al. 2007) such as *Bombyx mori* (Yukuhiro et al. 2002), *O. nubilalis and O. furnacalis* (Coates et al. 2005), *Adoxophyes honmai* (Lee et al. 2006), *Coreana* (Kim et al. 2006), *Antheraeapernyi* (Liu et al. 2008), *B. mandarina* (Pan et al. 2008), *Ochrogaster* (Salvato et al. 2008), *Artogeia melete* (Hong et al. 2009), *Eriogyna pyretorum* (Jiang et al. 2009), and *Hyphantria cunea* (Liao et al. 2010).

Nine PCGs had the common stop codon TAA, while the *nad2, cox1, cox2, nad4* have single T as an incomplete stop codon, also found in other animal mitochondrial genes (Clary and Wolstenholme 1985). The common interpretation of this phenomenon is that the TAA terminator is created via post– transcriptional polyadenylation (Ojala et al. 1981).

### Transfer and ribosomal RNA genes

**Figure 1.**
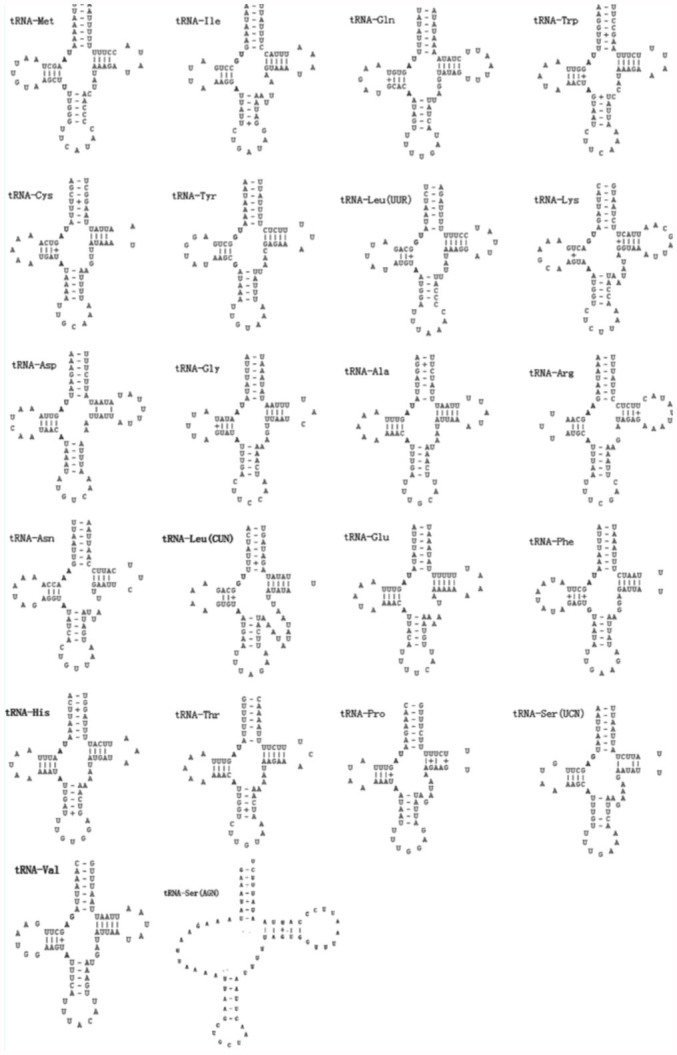
Putative secondary structures for the tRNA genes of the *Corcyra cephalonica* mitogenome. High quality figures are available online.

The 22 tRNA genes ranging from 64 to 73 nucleotides were spread over the mitogenome. Fourteen tRNAs were coded on the J-strand and eight on the N-strand, which is the same organization observed in other lepidopteran mitogenomes. Complete cloverleaf secondary structures could be inferred for 21 of the 22 tRNAs with the exception of *trnS1(AGN),* which lacks the DHU arm (Figure 1). A total of 43 unmatched base pairs were scattered in 20 tRNA genes, including 20 pairs in the DHU stems, eight pairs in the amino acid acceptor stems, nine pairs in the T^ψ^C stems, and six pairs in the anticodon stems. 24 of them are G-U pairs, which form a weak bond. The remaining were A-A, C-A, C-U, G-A, G-G, and U-U mismatches.

As in the other insect mitogenome sequences, two rRNA genes were present in *Corcyra.* The *rrnL* were found between *trnL(CUN)* and *trnV,* and the *rrnS* between *trnV* and the A+T rich region, respectively.

### Codon usage

Relative synonymous codon usage values of *Corcyra* mitogenome are summarized in Table 4. The codons CTG, CCG, and AGG were not represented in the coding sequences. Leucine (14.42%), isoleucine (12.14%), phenylalanine (9.74%), and serine (9.23%) were the most common amino acids in *Corcyra* mitochondrial proteins (45.53%). These amino acids are abundant in other insects, averaging 45.08% (Lessinger et al. 2000).

### Non-coding and overlapping region

The *Corcyra* mitogenome harbored 15 non-coding regions, from 1 to 351 bp to 512 bp. Intergenic spacer sequences covered four major regions of length more than 10 bp. The remaining intergenic spacer were less than 5 bp.

Spacer 1 (61 bp), located between *trnQ* gene and *nad2* gene, is a common intergenic spacer rich in AT nucleotides (96.72%). The location of this spacer is fixed in lepidopterans, but varied in length from 40 bp *(Parnassius bremeri)* (Kim MI 2009) to 88 bp *(Sasakia charonda)* (Unpublished, AP011824). This spacer can be taken as a lepidopteran mitogenome marker not found in other insect mitogenomes. Kim MI (2009) found that the intergenic spacer sequences and the *nad2* gene had higher sequence identity than other fragments of the mitogenome. There were 29 species with more than 60% identity of 32 total lepidopteran species sequenced (Table 5), suggesting that this spacer sequence originated from a partial duplication of the *nad2* gene.

Spacer 2 (49 bp) was found between *trnE* and *trnF* genes, including two microsatellite-like regions, (TA) 18 and (TTAT)3, similar to other lepidopterans. The spacer in *Adoxophyes* (Lee et al. 2006) is 222 bp and contains a different motif (TATTA)31. The spacer in *Ochrogaster* (Salvato et al. 2008) is 70 bp, contains a microsatellite (TA)23, and shows triplication of a 10-nucleotide motif with some changes. In other lepidoptera insects it is shorter than 10 bp.

**Figure 2.**
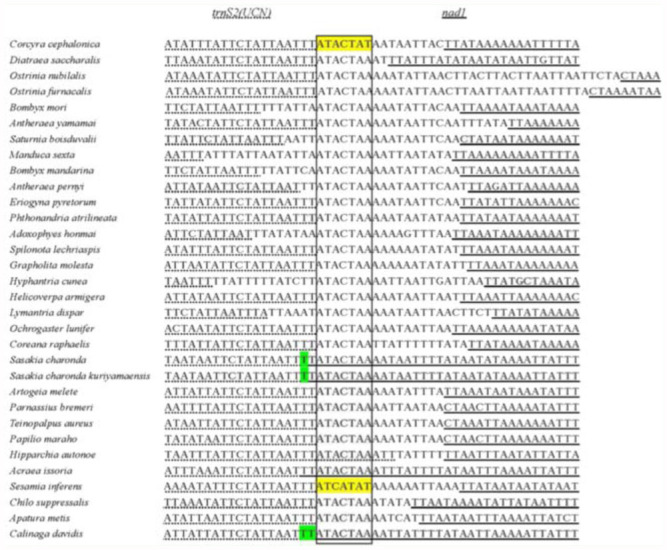
Alignment result of Spacer 4 tRNA-Ser(UCR)-NADI in 28 lepidopteran species. High quality figures are available online.

Spacer 3 (16 bp) was between the *trnS2(UCN)* and *nad1* genes, commonly detectable in lepidopteran insects, and measured 16–38 bp. This intergenic spacer sequence of most lepidopterans harbored the motif (ATACTAA), except for ATACTAT in *Corcyra* and ATCATAT in *Sesamia* (Figure 2). Similarly, in Hymenoptera there is a 6 bp conserved motif (THACWW) (Wei et al. 2010), and in Coleoptera the motif is 5 bp (TACTA). Such conservation suggests that the motif is functional in Lepidoptera. This motif is possibly fundamental to site recognition by the transcription termination peptide (Taanman 1999).

Spacer 4 (10 bp) was between *nad1* and *trnL(CUN). Ostrinia furnacalis* and *O. nubilalis* also showed 10 bp spacers, while other lepidopteran spacers measured 1–6 bp.

Spacer 5 (351 bp) was A+T rich and found between *rrnS* and *trnM* with AT nucleotides (96.58%). There was a motif ATAGA followed by a 20 bp poly-T stretch downstream of *rrnS,* and two microsatellitelike regions (TA)9 and (TA)8. Finally, a 10 bp poly-A was present upstream of *trnM.* The feature was found to be common for other lepidopterans sequenced to date.

Overlapping sequences had a total of 35 bp spread over eight regions. Like other insect species *(Adoxophyes)* (35), *atp8* and *atp6* had a seven-nucleotide overlap (ATGATAA), known to be translated from the same cistronic mRNAs. The longest overlapping sequence (8 bp) was between *trnW* gene and *trnC* genes. The remaining overlapping sequences were all less than 6 bp.

## Discussion

The *Corcyra* mitogenome is shorter than most lepidoteran mitogenomes previously reported. The shortest mitogenome is 15,140 bp *(Artogeia)* (Hong et al. 2009), and the longest is 15,928 bp *(B. mandarina)* (Pan et al. 2008). The *Corcyra* mitogenome had gene content and organization similar to other lepidopterans, which suggests that the mitochondrial gene arrangement in lepidopterans evolved independently after splitting from its stem lineage (Kim et al. 2006).

The most frequent amino acids in the *Corcyra* mitochondrial proteins were leucine, isoleucine, phenylalanine, and serine, all with high AT mutational bias that is a seemingly common feature in lepidopterans. Abascal et al. (2006) indicated that several arthropods have a new genetic code that translates the codon AGG as lysine instead of serineor arginine, these AGG reassignments may be events of parallel and correlated evolution between the arthropod genetic codes and the *trnK*/*trnS.* However, the variant codon, AGG, was not used by *Corcyra.*

The putative start codons of PCGs of the *Corcyra* mitogenome are ATNs, except for the CGA start codon of the *cox1* gene. Although tetranucleotides TTAG and hexanucleotide TATTAG have also been proposed as start codons for the *cox1* gene (Yukuhiro et al. 2002; Kim et al. 2006; Liu et al. 2008; Salvato et al. 2008; Kim SR et al. 2009), the TTAG lacks absolute conservation and may be of alternative function, not as an initiation codon (Margam et al. 2011). There are studies using ESTs (expressed sequence tags) to determine the *cox1* start codon. For example, some dipterans have an unorthodox UCG serine initiation codon, which was confirmed through mitogenome EST data (Morlais and Severson 2002; Krzywinski et al. 2006; Stewart and Beckenbach 2009). Mitogenome ESTs and alignment of the mitogenome sequence from all lepidopterans had shown that arginine (CGR) functions as the start codon of the *cox1* gene (Margam et al. 2011). These observations suggest that the use of EST data is valuable for the annotation of mitogenomes. The success of mitogenome sequencing will serve as the basis of the mating of EST and functional mitochondrial genome annotations.

**Table 1.**
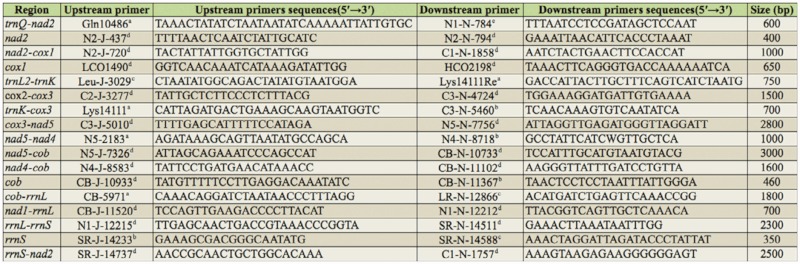
Region, primers and sequences for PCRs in this study.

**Table 2.**
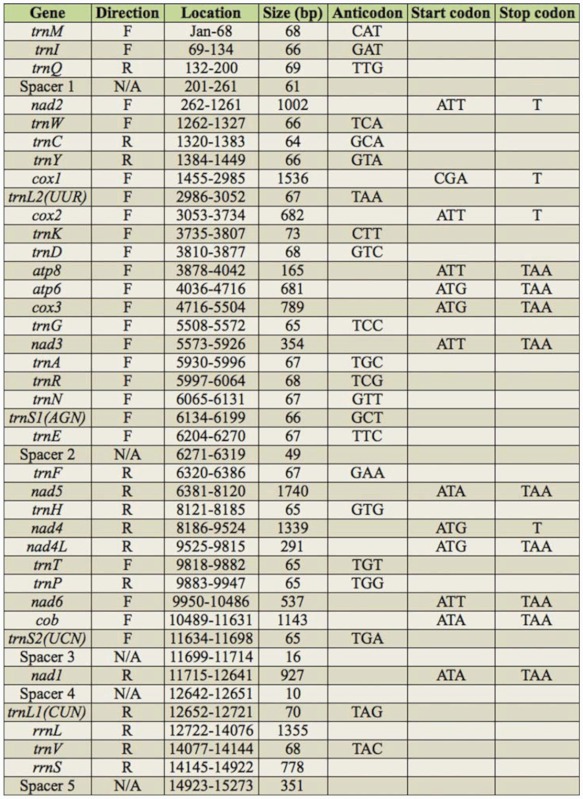
Summary of the mitogenome of the *Corcyra.*

**Table 3.**
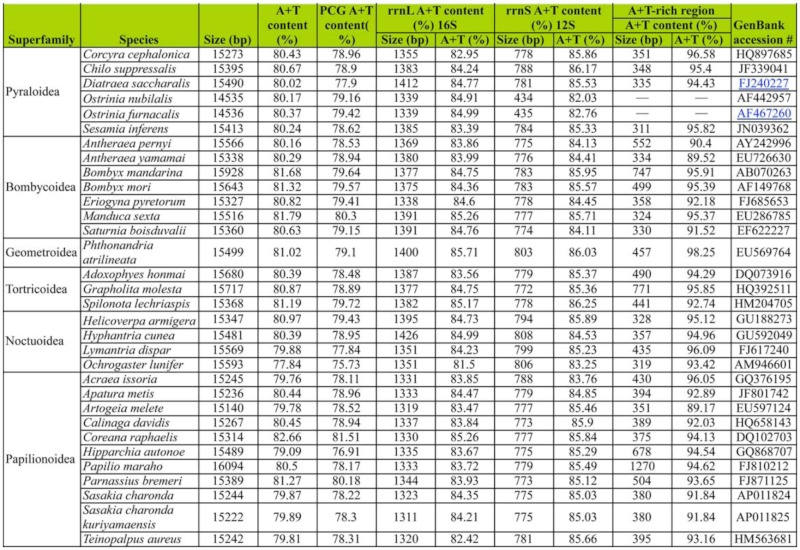
Characteristics of the lepidopteran mitogenomes.

**Table 4.**
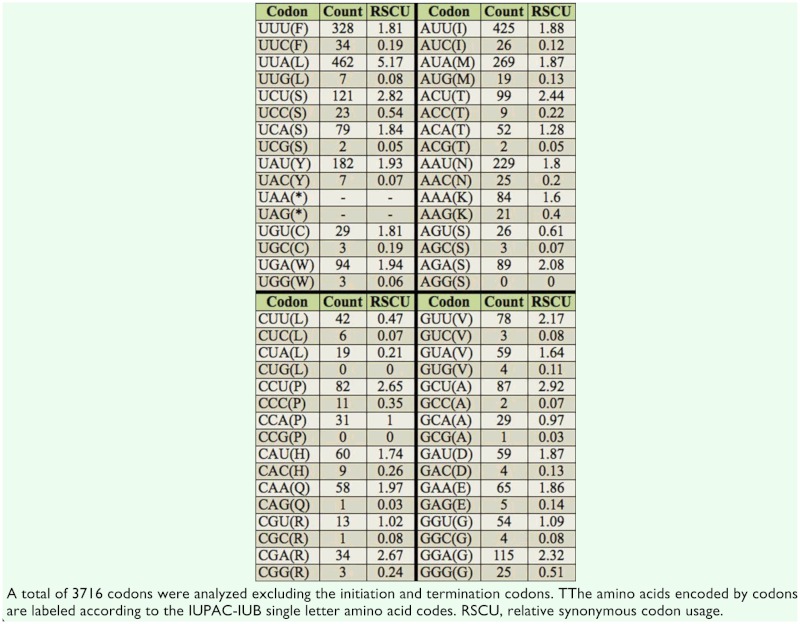
Codon usage in the *Corcyra* mitochondrial genome.

**Table 5.**
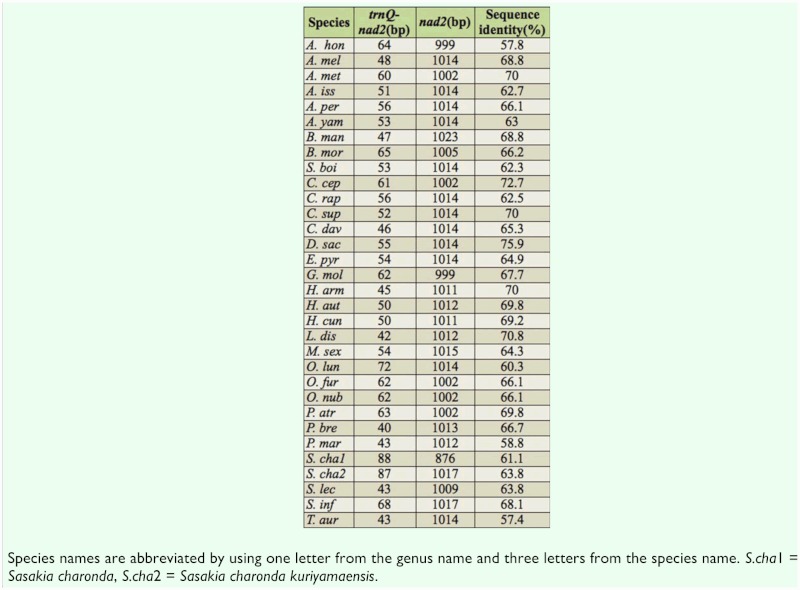
Sequence Identity of Spacer1 and *nad2* in 32 Lepidoptera species.

## **Abbreviations**

mitogenome,: mitochondrial genome;
PCGs,: protein-coding genes
